# Some pitfalls in application of functional data analysis approach to association studies

**DOI:** 10.1038/srep23918

**Published:** 2016-04-04

**Authors:** G. R. Svishcheva, N. M. Belonogova, T. I. Axenovich

**Affiliations:** 1 Institute of Cytology and Genetics, Siberian Branch of the Russian Academy of Sciences, Novosibirsk, Russia; 2 Vavilov Institute of General Genetics, the Russian Academy of Sciences, Moscow, Russia; 3 Novosibirsk State University, Novosibirsk, Russia

## Abstract

One of the most effective methods for gene-based mapping employs functional data analysis, which smoothes data using standard basis functions. The full functional linear model includes a functional representation of genotypes and their effects, while the beta-smooth only model smoothes the genotype effects only. Benefits and limitations of the beta-smooth only model should be studied before using it in practice. Here we analytically compare the full and beta-smooth only models under various scenarios. We show that when the full model employs two sets of basis functions equal in type and number, genotypes smoothing is eliminated from the model and it becomes analytically equivalent to the beta-smooth only model. If the basis functions differ only in type, genotypes smoothing is also eliminated from the full model, but the type of basis functions used for smoothing genotype effects becomes redefined. This leads to misinterpretation of the results and may reduce statistical power. When basis functions differ in number, no analytical comparison of the full and beta-smooth only models is possible. However, we show that the numbers of basis functions set unequal can become equal during the analysis, and the full model becomes disadvantageous.

Rapid progress in next generation sequencing technologies provides new opportunities for detection of rare genetic variants that control complex traits. However, statistical methods using single-variant association tests that are commonly adopted in genome-wide association studies are generally underpowered for rare variants. The statistical power of association analysis increases when the genetic variants in a genomic region are tested all at once, not individually^1^,^2^.

One of the most powerful regional mapping methods is based on functional data analysis (FDA)^3^,^4^. FDA is normally used for the continuous functional description of sets of discrete real data, for example, raw longitudinal phenotypes. The main rationale of using FDA in this case is reduction of the influence of noise and/or observation errors^5^. FDA has also been introduced into linear regression analysis. Functional linear regression models belong to a class in which the predictors are functions and the responses are scalars^6^,^7^,^8^. This class of models has been applied to regional associations analysis as an alternative to standard multiple regression models^9^. With FDA, the predictors of the regression models, namely, the spectrums of the discrete regional genotypes of each individual are described by continuous functions. The scalar responses of the regression models are defined as individual trait values. The effect of predictors is defined by a set of regression coefficients for the standard multiple regression or by a continuous function for the functional linear regression.

FDA can use less model parameters than the standard multiple regression, and, as a result, decrease the degrees of freedom of the statistical tests. FDA reduces the influence of noise and/or observation errors^5^. In genetic association analysis, functional linear models, unlike standard multiple linear regression models, utilize more detailed information on linkage disequilibrium because they consider not only the genotypes of multiple genetic variants within a particular region, but also the physical locations of these variants, that is, the order of these variants and the distances between them^3^,^9^.

With FDA, the genotypes of multiple genetic variants for each individual are described by a smoothing genetic variant function (GVF), while the effect of multiple genetic variants on a particular trait is described by a beta smoothing function (BSF)^3^,^9^. To build a smoothing function, a basis function system defined as a finite set of *K* standard independent mathematical functions should be set. Two basis function systems, B-spline and Fourier, are widely employed in regional association analysis^3^,^10^.

Although both GVFs and BSF have been introduced into FDA-based association analysis, only BSF is of interest for gene mapping research because statistical hypotheses are stated in terms of betas. To smooth genotype effects (betas), but not the genotypes themselves, a simplified version of the model, i.e., beta-smooth only, was proposed^3^,^4^,^10^,^11^. The statistical properties of the full and beta-smooth only versions of the functional regression models have been estimated under different scenarios using both independent and family-based samples^3^,^4^,^10^,^11^. Under these scenarios, the power of the beta-smooth only model was very close to that of the full model. Figure 1 illustrates this finding for power estimates that we obtained from analysis of the GAW17 family-based data set^12^, when both rare and common genetic variants were used for trait simulation and association analysis. We obtained the same results under scenarios that included only rare variants, different proportions of causal variants and unidirectional effects^10^. The same results were obtained in other studies using independent samples^3^,^4^,^11^,^13^. Moreover, for most genes tested on real data, the p-values calculated under the full and beta-smooth only models were identical (see, for example, Table 2 in ref. 13).

Questions arise: Are the full and beta-smooth only models equivalent and is it necessary to functionally represent genotypes when analyzing the association between a particular trait and multiple genotypes. To address these questions, we define a functional linear mixed model, to test the association using both independent and structured samples, and analytically consider scenarios that differ by the type and number of basis functions used to model GVFs and BSF.

## Models

The traditional linear regression model of multiple additive effects for an arbitrarily structured sample of *n* individuals is expressed as:


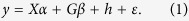


Here *y* is an (*n* × 1) known vector of trait values; *X* is an (*n* × *c*) known matrix of *c* covariates including a column of 1’s for the intercept; *α* is a (*c* × 1) unknown vector of fixed regression coefficients measuring the effects of *c* covariates; *G* is an (*n* × *m*) known matrix of genotypes of *m* genetic variants in the region, where *G*_*ij*_ is coded by the number of minor alleles of the *j*th genetic variant in the *i*-th individual; *β* is an (*m* × 1) unknown vector of fixed regression coefficients measuring the effects of *m* genotypes, and so *Gβ* is the regional genotypic component of the trait; *h* is an (*n* × 1) random vector of polygenic effects distributed as 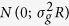, and *ε* is an (*n* × 1) random vector of errors distributed as 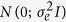, where 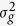 and 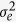 are the respective components of total variance 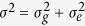 of the trait. Here *R* and *I* are the 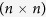 relationship and identity matrices, respectively. Model (1) assumes that the phenotypes *y* follow a multivariate normal distribution with a mean vector 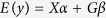 and a covariance matrix 
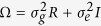
. If the sample consists of unrelated individuals then *R* = *I* and Ω = *σ*^2^*I*.

We further introduce a functional linear mixed model, which provides functional smoothing of both the genotypes and their effects on the trait:


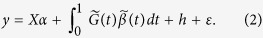


Here, 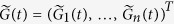 denotes an 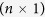 unknown vector of continuous genetic variant functions (GVFs), and 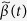 denotes an unknown continuous beta smoothing function (BSF) under *t*. In actual data, *t*_1_,…,*t*_*m*_ are the ordered physical positions of genetic variants in the region [*t*_1_, *t*_*m*_]. We scale [*t*_1_, *t*_*m*_] to [0, 1] and let *t* be a real number in [0, 1] that defines the position of a particular genetic variant in the scaled region. By applying FDA, GVFs and BSF can be described by sets of *K*_*G*_ and *K*_*β*_ basis functions, respectively. Then, according to^10^, 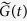 and 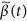 are estimated as


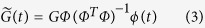


and


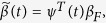


where *ϕ*(*t*) = (*ϕ*_1_(*t*),…,*ϕ_K__G_*(*t*))^*T*^ is a (*K*_*G*_ × 1) vector of basis functions, which were used to smooth the genotypes; *Ф* is an (*m* × *K*_*G*_) matrix with element *Ф*_*ij*_ = *ϕ*_*j*_(*t*_*i*_); *ψ*(*t*) = (*ψ*_1_(*t*),…,*ψ_K__β_*(*t*))^*T*^ is a (*K*_*β*_ × 1) vector of basis functions, which were used to smooth the genotype effects; and, finally, 
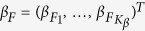
 is a (*K*_*β*_ × 1) vector of regression coefficients.

Substituting the expressions for 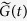 and 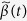 from (3) to equation (2) yields


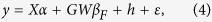


where


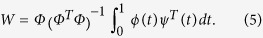


The (*m* × *K*_*β*_) smoother-matrix *W* is constructed from two sets of basis functions, *ϕ*(*t*) and *ψ*(*t*), intended for smoothing genotypes and their effects, respectively. Matrix *W* depends on the type and number of the given basis functions, as well as on the positions of genetic variants in the region. Models (1) and (4) differ by regional genotypic components: *Gβ* versus *GWβ*_*F*_. Moreover, the parameters associated with genotype effects appear as vector *β*_*F*_ of size (*K*_*β*_ × 1) in model (4) and as vector *β* of size (*m* × 1) in model (1).

A simplified functional linear model, which does not smooth genotypes, can be constructed by discretization of the full functional linear model (4) (Chapter 15 in ref. 5). In this case, only beta smoothing function 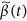 is used with set *ψ*(*t*) of 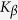 basis functions:


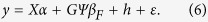


In model (6), *Ψ* is an (*m* × *K*_*β*_) smoother-matrix constructed similarly to the (*m* × *K*_*G*_) matrix *Φ* from model (4), i.e., *Ψ*_*ij*_ = *ψ*_*j*_(*t*_*i*_); so the matrix *Ψ* depends on the set of basis functions and the positions of the genetic variants. Model (6) is called beta-smooth only^3^,^4^,^11^,^13^.

## Test statistics

To test for an association between the genomic region and the trait, we test null hypothesis *H*_0_: 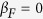 against alternative hypothesis *H*_1_: 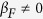 with test statistics using the residual sums of squares (RSS). For example, this test statistics could be^5^,^14^


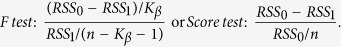


Here 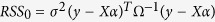 and 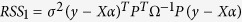 are the sums of the squares of residuals under *H*_*0*_ and *H*_*1*_, respectively; Ω, *σ*^2^ and *α* are estimated under *H*_*0*_ and *P* is a projection matrix given as


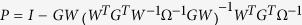


and


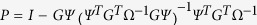


for models (4) and (6), respectively.

### Comparison of the different models

A comparison of expressions (4) and (6) indicates that regional genotypic components of trait *y* under the models have similar forms: either *GWβ*_*F*_ or *GΨβ*_*F*_. In these expressions, *G* denotes the matrix of real genotypes, *β*_*F*_ is a vector of *K*_*β*_ estimated regression coefficients, and *W* and *Ψ* are the (*m* × *K*_*β*_) smoother-matrices. These transforming matrices allow the genotypes of *m* variants with *K*_*β*_ regression coefficients to be combined to calculate the regional genotypic component of the trait. The parameters of interest in both models are the regression coefficients *β*_*F*_. When the numbers, *K*_*β*_, of the coefficients are equal in two models, the degrees of freedom of statistical tests in these models are equal, too. For the *F*-test, df_1_ = *K*_*β*_ and df_2_ = *n *− *K*_*β*_ − 1, and the score test is approximated by the χ^2^ distribution with df = *K*_*β*_.

Models (4) and (6) differ in smoother-matrix construction. Smoother-matrix *W* in model (4) is defined by two sets of basis functions, namely, *ϕ*(*t*) and *ψ*(*t*). By contrast, smoother-matrix *Ψ* in model (6) uses only one basis function set, *ψ*(*t*). Formally, we cannot trace whether the genotypes, or their effects, or both are smoothed in each model, because any smoother-matrix is simply a transforming matrix constructed using the set(s) of basis functions and the positions of the genetic variants. Therefore, the biological meaning attributed to the smoothing process is lost when the models are formally described by expressions (4) and (6).

The regression coefficients (beta-parameters) of models (4) and (6) are estimated using the maximum likelihood approach as:


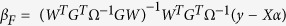


and


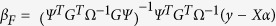


respectively.

With these estimates, it is easy to calculate the regional genotypic component of trait *y* defined by models (4) and (6) as:


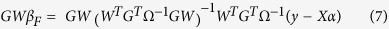


and


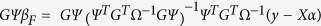


respectively.

To compare models (4) and (6), we present *W* in (5) as the product of three matrices, *W*_1_, *W*_2_, and *W*_3_:


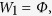



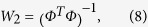


and


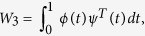


of dimensions 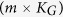, (*K*_*G*_ × *K*_*G*_) and (*K*_*G*_ × *K*_*β*_), respectively. Unlike matrices *W*_1_ and *W*_2_, matrix *W*_3_ is independent of the actual data, and is defined only by sets of basis functions *ϕ*(*t*) and *ψ*(*t*).

Expression (7) describing the regional genotypic component of trait *y* in model (4) can be rewritten in terms of matrices *W*_1_, *W*_2_ and *W*_2_ as:


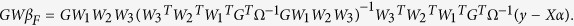


Note that matrix *W*_2_ is always invertible, while the invertibility of matrix *W*_3_ depends on how *K*_*G*_ and *K*_*β*_ relate to each other. Here, only two situations are possible: *K*_*G*_ = *K*_*β*_ and *K*_*G*_ > *K*_*β*_, because the number of basis functions for GVFs should not be less than that for BSF^10^.

### *K*_*G *_= *K*_*β*_ situation

If *K*_*G*_ = *K*_*β*_, matrix *W*_3_ is invertible (see (8)). Therefore, matrices *W*_2_ and *W*_3_ can be canceled in expression (9). Therefore, *GWβ*_*F*_ is expressed only in terms of *W*_1_, *G*, and Ω, i.e., using (8) in terms of *Φ*, *G*, and Ω:


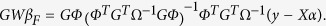


Hence, when *K*_*G*_ = *K*_*β*_, model (4) is defined by only one set of basis functions *ϕ*(*t*), and does not use the second set, *ψ*(*t*). In this situation model (4) simplifies to model (6), where *Ψ*_*ij*_ = *ϕ*_*j*_(*t*_*i*_).

**Figure 1:**
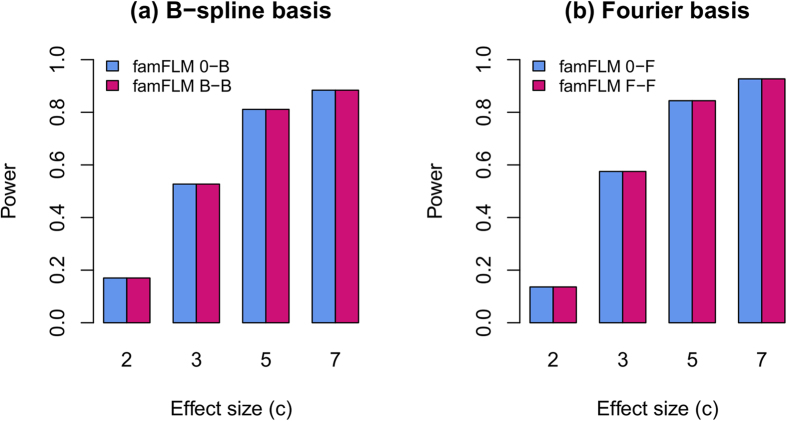
Statistical power of the FDA-based regional association analysis of familial data (famFLM). Compared functional models are: (**a**) the beta-smooth only model using B-spline basis for BSF (0-B) and the full model using B-spline basis for both GVFs and BSF (B-B); (**b**) the beta-smooth only model using Fourier basis for BSF (0-F) and the full model using Fourier basis for both GVFs and BSF (F-F). Power was estimated as a proportion of *P* ≤ 2.5 × 10^−6^. A region with 50 genetic variants from the GAW17 dataset^12^ was used for simulation. 10% of variants were randomly selected as causal. For each causal variant, the effect size was defined as *β* = ln(*c*) |log_10_(*MAF*)|/2 (constant *c* is shown along the horizontal axis).

In particular, if model (4) employs two sets of basis functions identical in type and number, then *ψ*(*t*) = *ϕ*(*t*) and model (4) just reduces to its simplified version (6). In mathematical terms, the model with “double” smoothing becomes equivalent to that with “single” smoothing. In terms of biological meaning, the model with both genotypes and betas smoothed becomes equivalent to that with beta smoothing only. A comparison of expressions (4) and (6) demonstrates that the equivalence of models (4) and (6) is explained by the equivalence of regional genotypic components of the trait. Although the BSFs under models (4) and (6) may be different, as is shown in ref. 11, Fig. 1, the regional genotypic components remain identical. A majority of published studies that compare the statistical power of the full and beta-smooth only models use two sets of basis functions equal in type and number. In light of our results, it becomes clear that the similarity of power estimates for two models is explained by their analytical equivalence rather than by numerical similarity.

**Figure 2:**
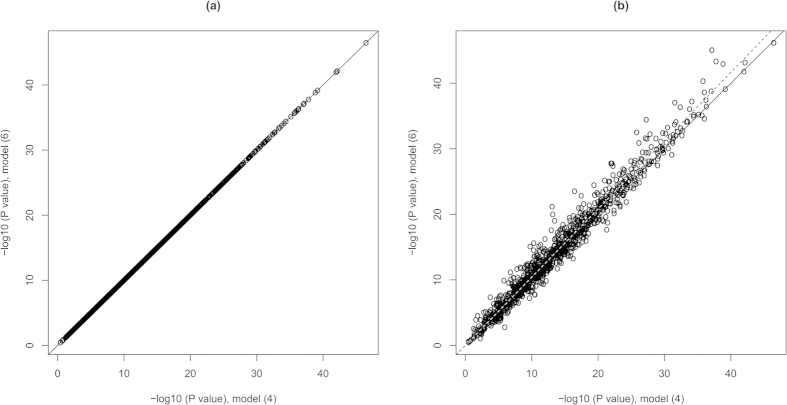
Comparison of model (4) using B-spline basis for GVFs and Fourier basis for BSF with model (6) using B-spline (**a**) or Fourier (**b**) basis for BSF. *K*_*G*_ = *K*_*β*_ = 25. For two models compared in panel (**b**), powers estimated as a proportion of *P* ≤ 2.5 × 10^−6^ were 0.861 and 0.876 for the full and beta-smooth only models, respectively. The solid line indicates a one-to-one correspondence; the dotted line is the linear regression line. The same data as in Fig. 1 were used.

If model (4) employs two sets of basis functions that differ only in their type then *ψ*(*t*) ≠ *ϕ*(*t*) and model (4) reduces to model (6), in which the type of basis functions is *ϕ*(*t*) rather than *ψ*(*t*). In terms of biological meaning, the set of basis functions selected for genotype smoothing in the full model switches to beta smoothing. In this case, the full model may be misleading and/or underpowered. For example, betas are expected to be smoothed by the Fourier basis if the B-spline and the Fourier bases are set for GVFs and BSF, respectively. The results of analysis are in fact equivalent to those obtained from beta-smooth only model with the B-spline basis employed (Fig. 2a). In this situation, the researcher is misled about the type of basis functions used for beta smoothing. If the researcher had initially used the beta-smooth only model with the Fourier basis (instead of the full model), the statistical power of analysis would have been increased (Fig. 2b).

In any case, the full model is unjustified at *K*_*G*_ = *K*_*β*_, for the same (or better) association analysis results can more easily be obtained using beta-smooth only model (6).

### ***K***_***G ***_> *K* *_β_* situation

**Figure 3:**
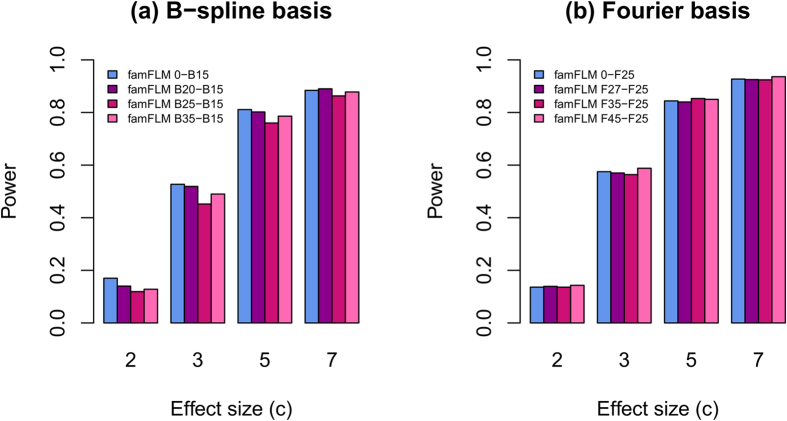
Statistical power of the beta-smooth only and full models when *K*_*G *_> *K_β_*. Notations are the same as in Fig. 1. Numbers in legend are the numbers of basis functions: for example, B20–B15 means that 20 and 15 B-spline functions were used for GVFs and BSF, respectively. The same data as in Fig. 1 were used.

Only when *K*_*G*_ > *K*_*β*_, matrix *W*_3_ is not invertible (because this matrix is not square) and cannot be canceled in expression (9). As a result, the statistics under models (4) and (6) are different. Although the degree of freedom remains the same for both models, their smoothing strengths may differ. It is not *a priori* clear which model will have a greater smoothing strength. Moreover, increase in smoothing strength does not always lead to increase in power. As a result, it is impossible to predict when the full model is more powerful than the beta-smooth only model. Figure 3 illustrates that power can change unpredictably when using the full model.

If the researcher still decides to use the full model with two sets of basis functions, the number of basis functions for GVFs and BSF should be controlled to ensure that *K*_*G*_ > *K*_*β*_. Otherwise, the full model may become disadvantageous, as we demonstrated previously. However, the number of basis functions defined by the researcher may be changed during analysis. Such situations occur due to trivial restrictions of FDA methods. In particular, the number of genetic variants in the region should not be less than the number of basis functions for GVFs, and the number of basis functions for GVFs should not be less than that for BSF, that is, *m *≥ *K*_*G*_ ≥ *K*_*β*_^10^. The available software packages for FDA-based association analysis reduce the number of basis functions for BSF to that for GVFs; that is, *K*_*β*_ becomes equal to *K*_*G*_ when the condition *K*_*G*_ ≥ *K*_*β*_ is not satisfied. When the genotype matrix includes linear-dependent genotype columns, the number of genetic variants analyzed in the region is reduced to ensure that the matrices are invertible. If the number of genetic variants is reduced to less than the declared *K*_*G*_ value, then this value automatically decreases to *m*. In this case, *K*_*β*_ may become equal to *K*_*G*_. The *K*_*G*_ value is difficult to control; therefore, the predictability of the behavior of the model with both GVFs and BSF decreases even in those rare cases, when certain advantages can be expected. On the other hand, smoothing strength can easily be regulated without GVFs by adjusting the *K*_*β*_ value in the beta-smooth only model.

In all cases, the full model increases the running time^10^, is less predictable and is more difficult to interpret when *K*_*G*_ > *K*_*β*_.

## Conclusions

We have demonstrated that there is no reason to use the full models that utilize equal sets of basis functions, because the same results can easier be obtained using beta-smooth only models. As far as the full models that use different sets of basis functions are concerned, we have identified several situations, in which genotype smoothing is counterproductive in that it may cause an unpredictable behavior of the model and reduce statistical power. Moreover, we have identified several situations, in which unequal numbers of basis functions defined by the researcher become equal during the analysis, and the full model becomes equivalent to the beta-smooth only model. Thus the full model offers only illusory benefits in practice. It has hidden pitfalls that should be taken into consideration in planning functional association analyses.

## Additional Information

**How to cite this article**: Svishcheva, G. R. *et al*. Some pitfalls in application of functional data analysis approach to association studies. *Sci. Rep*. **6**, 23918; doi: 10.1038/srep23918 (2016).
